# Effect of Static Load on the Nucleus Pulposus of Rabbit Intervertebral Disc Motion Segment in an Organ Culture

**DOI:** 10.1155/2016/2481712

**Published:** 2016-10-31

**Authors:** Jia-Wen Zhan, Min-Shan Feng, Li-Guo Zhu, Ping Zhang, Jie Yu

**Affiliations:** ^1^General Orthopedics Department, Wangjing Hospital, China Academy of Chinese Medical Sciences, Beijing 100102, China; ^2^Key Laboratory of Beijing of Palasy Technology, Wangjing Hospital, China Academy of Chinese Medical Sciences, Beijing 100102, China; ^3^Spine Department 2, Wangjing Hospital, China Academy of Chinese Medical Sciences, Beijing 100102, China; ^4^Pathology Department, Wangjing Hospital, China Academy of Chinese Medical Sciences, Beijing 100102, China

## Abstract

The development of mechanically active culture systems helps in understanding of the role of mechanical stress in intervertebral disc (IVD) degeneration. Motion segment cultures facilitate the application and control of mechanical loads. The purpose of this study was to establish a culturing method for rabbit IVD motion segments to observe the effect of static load on the whole disc organ. Segments were cultured in custom-made apparatuses under a constant, compressive load (3 kg) for 2 weeks. Tissue integrity, matrix synthesis, and matrix gene expression profile were assessed and compared with fresh one. The results showed* ex vivo* culturing of samples gradually destroyed the morphology. Proteoglycan contents and gene expression were decreased and downregulated obviously. However, immunohistochemical staining intensity and collagen type II gene expression were significantly enhanced and upregulated. In contrast, these trends were reversed under constant compression. These results indicated short-term static load stimulated the synthesis of type II collagen; however, constant compression led to progressive degeneration and specifically to proteoglycan. Through this study a loading and organ-culturing system for* ex vivo* rabbit IVD motion segments was developed, which can be used to study the effects of mechanical stimulation on the biology of IVDs and the pathomechanics of IVD degeneration.

## 1. Introduction

Evidence of a link between degenerative intervertebral disc (IVD) and low back pain (LBP) is mounting [1]. Currently, the treatment of diseases related to IVD often involves surgical intervention or long-term rehabilitation therapy. The goals of biological therapy are to prevent or delay IVD degeneration and to alleviate its symptoms by promoting tissue repair. To better prevent and treat LBP a detailed understanding of the mechanisms of IVD degeneration is necessary.

The comprehensive mechanisms and related biological and mechanical pathways of IVD degeneration remain poorly understood [2, 3], even though LBP is a common clinical condition. Biomechanics and IVD degeneration are closely related, and many studies have shown that mechanical loading is one of the major factors leading to IVD [4, 5]. An epidemiological survey has also shown that mechanical loading is a risk factor for LBP [6, 7]. However, weight-bearing mechanical loading is the primary function of IVDs, and it occurs in the natural environment. Mechanical loading also stimulates IVD cells; this stimulation is likely the underlying factor for the maintenance of cartilage cell activity [8, 9]. To better understand the mechanisms of IVD degeneration, an in-depth understanding of its cause and effect relationship with biomechanics is required. Moreover, the establishment of an ideal experimental model might facilitate future related studies.

However, investigating the complex metabolism and signaling cascades that occur in IVDs is difficult using* in vivo* models [10] because of the lack of close control and difficulties with monitoring. In contrast,* ex vivo *culturing systems are more appealing because they allow for better control of biochemical and biomechanical factors [1]. Therefore,* ex vivo *organ models represent an experimental basis to observe the responses and changes of IVD tissues to specific external stimuli [1, 9–22], such as mechanical loads [23, 24] or biologics [25, 26]. Thus, an IVD motion segment was established and maintained in an organ culture [27–29], and it included the whole IVD organ and adjacent vertebral bodies (VBs). The motion segment model has certain advantages. First, it enables the* ex vivo *organ model to be more reflective of the true conditions. Second, the adjoining VBs (as rigid fixtures) facilitate the application and control of complex mechanical loads [27].

This study compared rabbit IVD motion segments cultured in custom-made apparatuses under static load with fresh tissues. The tissue integrity, matrix synthesis, and matrix gene expression profile were assessed to determine the influence of static load on the IVDs and to increase understanding of these conditions so that the effects of biomechanics on the IVDs can be better controlled.

## 2. Materials and Methods

### 2.1. Ethics Statement

All of the experimental procedures were conducted in accordance with the institutional guidelines for the care and use of laboratory animals of the China Academy of Chinese Medical Sciences, Beijing, China. The experimental animals included 9 eighteen-week-old (3.0 kg) male New Zealand White rabbits. These animals were used in accordance with protocols that were approved by the animal ethics committee of the Institute of Basic Theory for Chinese Medicine, China Academy of Chinese Medical Sciences (approval number 20141002). The animals were anesthetized with pentobarbital (100 mg/kg). Five minutes before death, 25,000 IU heparin was administered through the ear vein, and the lumbar spine was harvested under sterile conditions immediately after euthanasia via CO_2_ asphyxiation.

### 2.2. Intervertebral Disc Motion Segment Culture Method

IVD motion segments were obtained from the 9 rabbits. Soft tissues and posterior elements of the spine were removed, and the motion segment models were dissected from consecutive levels (*n* = 4/animal) consisting of the whole disc organ, which included the vertebral end plates (EPs), annulus fibrous (AF), and nucleus pulposus (NP) with adjacent VBs. The lengths of the IVD motion segments ranged from 280 to 310 mm, and the diameters of the IVDs ranged from 110 to 140 mm. Any debris on the cut surfaces was washed with phosphate-buffered saline using a syringe with an 18-gauge needle; all of the IVDs were rinsed for 2 minutes in Hanks' Buffered Salt Solution (HBSS) containing heparin and 10% penicillin-streptomycin.

A total of 36 IVD motion segments were maintained in custom-made apparatuses under a constant compressive load (3 kg, 0.5 MPa). The apparatuses were maintained in an incubator under standard culture conditions (37°C, 5% CO_2_; Figure S1 in Supplementary Material available online at http://dx.doi.org/10.1155/2016/2481712).

The specimens were maintained in Dulbecco's Modified Eagle's Medium (DMEM) supplemented with 20% fetal bovine serum (FBS; Invitrogen Life Technologies, Carlsbad, CA, USA), 50 mg/mL L-ascorbate, 100 U/mL penicillin, 100 mg/mL streptomycin, and 2.5 mg/mL Fungizone. NaCl was added to the DMEM to raise the osmolarity to 410 mOsm/kg. The media were changed every two days. After 0, 3, 7, and 14 days, seven IVDs were assessed by hematoxylin and eosin (HE) staining, Alcian blue-periodic acid Schiff reaction (AB/PAS), and collagen type II immunohistochemistry (IHC), and real-time polymerase chain reaction (PCR) was performed to examine the matrix gene expression profile.

### 2.3. Loading and Culturing Apparatuses for the Intervertebral Disc Motion Segments

The apparatuses were designed for the* ex vivo *culturing of IVD motion segments while simultaneously providing static axial compression. They consisted of two components: a loading frame and a culture chamber.

The loading frame provided a static load through the use of weights on a mobile loading plate that could be moved along the optical axis. Because compression can cause the IVD heights to change slightly, the mobile weight ensured that the full load was being consistently applied to the specimen. A gland was fixed to the bottom of the loading plate, through which the weight was applied to the model. A gland also covered the top of the chamber to ensure that the culture environment was closed (Figure S2).

A disposable silicon tube was inserted at the bottom of culture chamber, and it functioned as an outlet for media changing; the other end of the tube was plugged. A syringe was used for changing the media, and the manipulations were performed on an aseptic operation table. Moreover, the tubing and needle were changed with each use to ensure sterility of the culture (Figure S2).

Based on the characteristics of the spinal motion segment, the device was arranged on the top and base pedestals. Three jackscrews were inserted through the pedestal to fix one side of the VBs, allowing the motion segment to remain in an upright state so that the compression was always vertically loaded on the surfaces of the IVDs (Figure S2).

All of the components could be disassembled from the device, and the materials were resistant to deformation and decomposition by disinfection treatments (e.g., high temperature and high pressure). Moreover, the whole apparatus was sufficiently small; thus, multiple loading stations could be placed in the incubator at the same time (Figure S1; patent number: ZL 201420568511.9).

### 2.4. Histological Analysis

The samples were fixed with a 10% buffered neutral formalin solution for up to 2 days, decalcified in ethylenediaminetetraacetic acid (EDTA), and then embedded in paraffin. Midsagittal sections were cut with thicknesses of 4 to 6 *μ*m. The sections were deparaffinized in xylene, rehydrated with graded ethanol, and stained with HE. Then, they were viewed by light microscopy.

AB/PAS staining was used to detect synthesized proteoglycans in the IVD tissues.

### 2.5. Collagen Type II Immunohistochemistry

For detection of the NP extracellular matrix, a type II collagen monoclonal antibody (Sigma, USA) was used for IHC analyses. Briefly, at each harvesting time point, 4 to 6 *μ*m paraffin sections from three samples were dewaxed, rehydrated, and then blocked with hydrogen peroxide as an endogenous peroxidase. Next, the sections were washed in dH_2_O and treated with 1 of 2 enzyme antigen retrieval reagents for 20 minutes at 37°C. They were subsequently washed again, and nonspecific binding sites were blocked at room temperature for 45 minutes with 20% w/v goat serum. Then, the sections were incubated with a mouse monoclonal primary antibody against type II collagen (Sigma-Aldrich, Bale, Switzerland) diluted 1 : 20 overnight at 4°C. Negative controls were used for which the primary antibody was replaced with an equal concentration of mouse IgG (Sigma). After the sections were again washed, those being assessed for collagen type II were incubated with biotinylated rabbit antimouse antiserum (Sigma) diluted 1 : 400 for 30 minutes at room temperature. Secondary antibody binding was visualized using the streptavidin-biotin complex technique with a 3,3′-diaminobenzidine tetrahydrochloride solution as the colored reaction product. The sections were counterstained with hematoxylin, dehydrated, and mounted. An NIS-Elements D2.30 image analysis system (Nikon, Tokyo, Japan) was used for semiquantitative analysis of type II collagen.

### 2.6. Real-Time PCR Analysis

Total RNA from the NP was analyzed for the expression of* ECM* genes. Briefly, the AF was cut with a blade, and the NP tissue was removed using a micro curette and immediately frozen in liquid nitrogen at each harvesting time point. Tissues from 3 IVDs were pooled to ensure that a sufficient quantity of tissues was obtained for RNA isolation [28, 30, 31], which was performed using Trizol reagent (Invitrogen Life Technologies, Carlsbad, CA, USA) according to the manufacturer's instructions. Primers for the rabbit* GAPDH* and* ECM* genes were custom designed using Primer 5.0 software (Applied Biosystems, Foster City, CA) (Table 1).

Quantitative real-time polymerase chain reaction (qRT-PCR) was performed using a SYBR Green One-Step qRT-PCR kit (KAPA Biosystems, USA). The final qRT-PCR results were obtained using the comparative Ct method with the following equation:

(1) $$\begin{array}{c} \text{Fold change}=2^{-\Delta \Delta \text{Ct}}, \end{array}$$

where −ΔΔCt = (Ct_target_ − Ct_reference_)_control_ − (Ct_target_ − Ct_reference_)_culture_. (reference: mean Ct of* GAPDH*; control: day 0; and culture: day 3, 7, 14, or 21).

### 2.7. Statistical Analyses

Statistical analyses, including analysis of variance (ANOVA) and* post hoc* pairwise comparisons, were performed using SPSS software (ver. 16.0, SPSS, Inc., Chicago, IL). The between-group data were compared using the independent-samples* t*-test. All of the results are expressed as the mean ± standard deviation (SD). Statistical significance was set at *P* < 0.05.

## 3. Results

### 3.1. Histological Analysis

**Figure 1:**
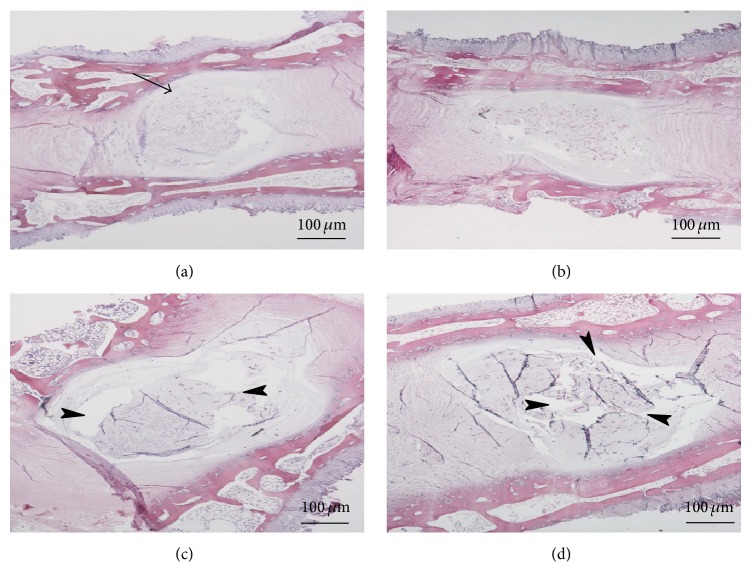
HE staining of IVD midsagittal slices at each time point under static load conditions (original magnification, ×10). (a) Fresh tissue; (b, c, d) histological observations of tissues under static load on days 3, 7, and 14, respectively. In the fresh tissue, the tissue structure was intact (arrows) (a). Under static load, culturing to 7 days (c) led to a decrease in the number of NP cells and their dispersal (arrowheads). Culturing for 14 days (d) led to the loss of the tight concentric architecture of the AF and separation of the NP (arrowheads); this change was more obvious under the static load than fresh tissue. HE: hematoxylin-eosin; IVD: intervertebral disc; AF: annulus fibrous; NP: nucleus pulposus.

Prior to culturing, HE staining showed that the central integrated NP contained abundant cells and matrix material. In addition, the AF was arranged in concentric circles with a clear hierarchy and clear boundaries between the NP and the cartilage EP at the upper and lower ends. Under static load, the integrity of the tissue was maintained for 3 days. Culturing for 7 days led to a decrease in the number of NP cells and their dispersal, as well as partial laceration of the AF. Culturing for 14 days led to loss of the tight concentric architecture of the AF and separation of the NP; this change was more obvious under static load than the fresh tissue (Figure 1).

**Figure 2:**
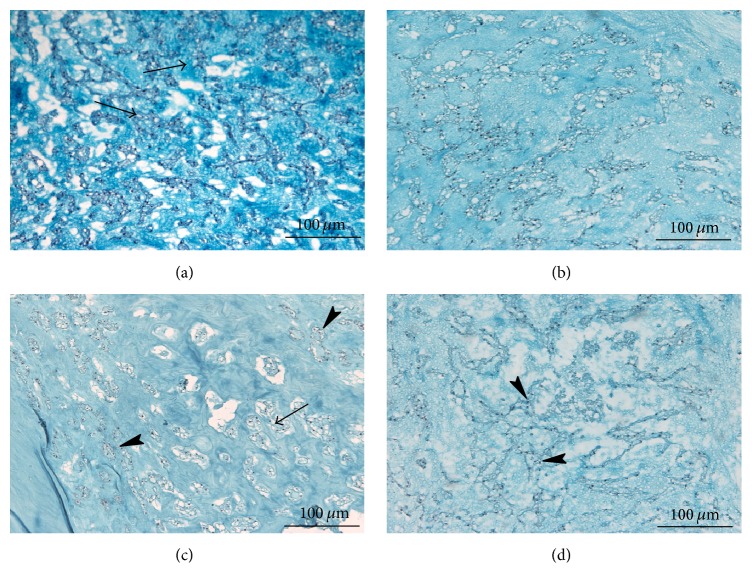
AB/PAS staining of proteoglycans in the NP at each time point (original magnification, ×200). (a) Fresh tissue; (b, c, d) AB/PAS staining of tissues under static load on days 3, 7, and 14, respectively. The staining within the matrix and in the immediate pericellular areas indicated the presence of proteoglycans (a, arrows). Under static load, by 7 days (c), the proteoglycan content decreased (arrow) and was unevenly distributed (arrowheads). By 14 days (d), the number of cells in the NP obviously decreased (arrowheads). AB/PAS: Alcian blue and periodic acid-Schiff; NP: nucleus pulposus.

The specimens were stained with AB/PAS to observe changes in the proteoglycan content [10]. The staining within the matrix and in the immediate pericellular areas indicated the presence of proteoglycans. Under static load, the proteoglycan content decreased and was unevenly distributed by 7 days. Moreover, by 14 days, the number of cells in the NP was obviously decreased, although no further decrease in staining was observed, suggesting a reduction in biosynthetic activity (Figure 2).

### 3.2. Collagen Type II Immunohistochemistry

**Figure 3:**
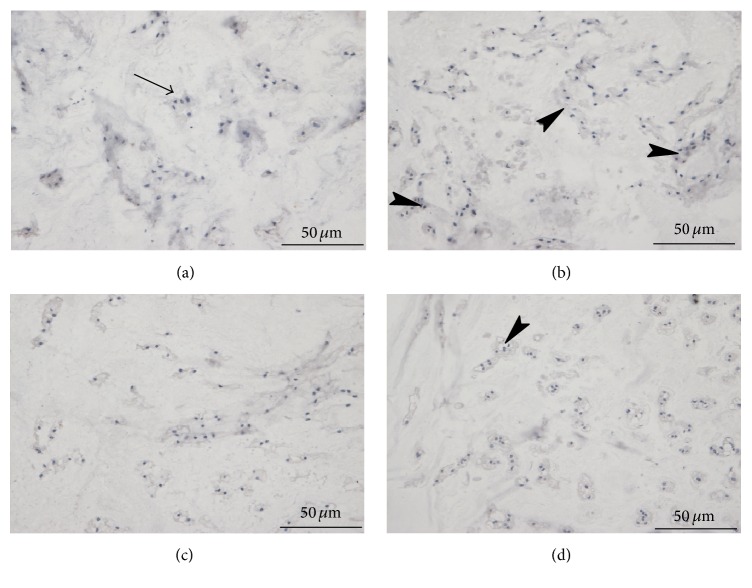
Collagen type II IHC staining of the NP at each time point (original magnification, ×400). (a) Fresh tissue; (b, c, d) collagen type II staining of tissues under static load at days 3, 7, and 14, respectively. The samples under static load showed a significant enhancement in IHC intensity for the initial 3 days (b, arrowheads); however, the staining was obviously reduced after 3 days; by 2 weeks (d), the staining was significantly decreased (arrow) and significantly different from the controls. IHC: immunohistochemistry; NP: nucleus pulposus.

**Figure 4:**
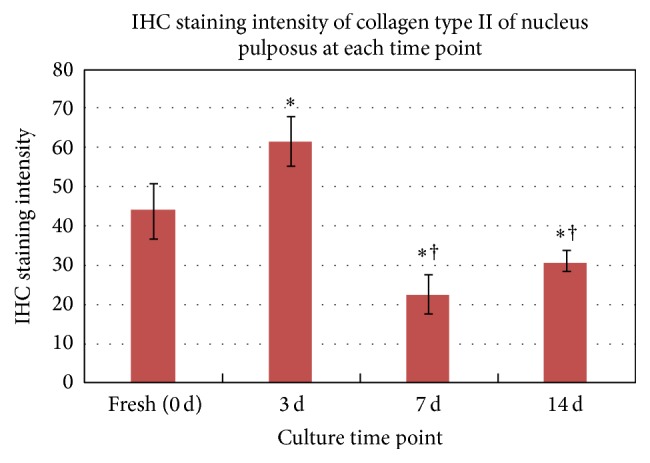
Collagen type II IHC staining intensity of the NP at each time point. The samples under static load showed a significant enhancement at 3 days, and significant differences were observed with fresh tissues. At 7 days, the staining was obviously decreased, and by 2 weeks, it was significantly decreased (*P* < 0.05) and significantly different from that of the fresh tissue. The values represent the means ± SDs. ^*∗*^ *P* < 0.05 versus day 0; ^†^ *P* < 0.05 versus day 3. IHC: immunohistochemistry; NP: nucleus pulposus; SDs: standard deviations.

Immunoreactive type II collagen was observed within the matrix of the NP at all times. The samples under static load showed a significant increase in IHC intensity during the initial 3 days, and significant differences were observed with fresh tissues (*P* < 0.05). However, the staining was obviously reduced after 3 days, and by 2 weeks, it was significantly decreased (*P* < 0.05) (Figures 3 and 4, Table 2).

### 3.3. ECM Gene Expression

**Figure 5:**
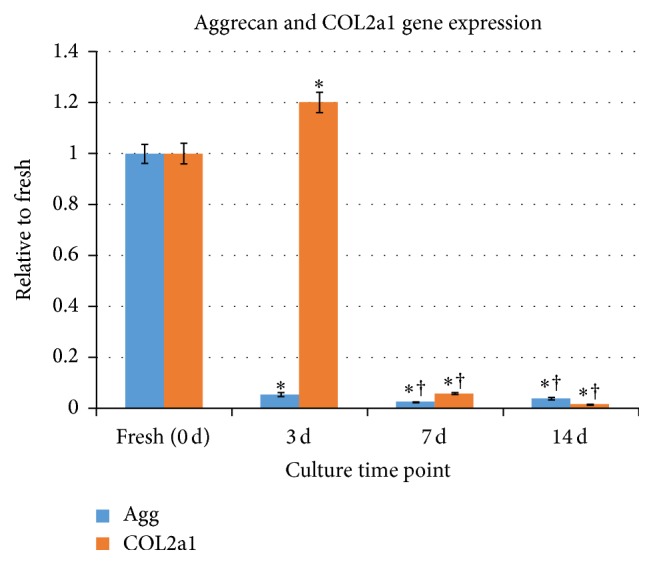
Relative expression quantified by real-time PCR. An obvious downregulation of* Agg* was observed under static load during culture compared with fresh tissue, whereas an upregulation of* COL2A1* was observed in response to static load by 3 days, and the expression of this gene subsequently decreased until day 14 to below detectable levels. The values represent the means ± SDs normalized to fresh tissue. ^*∗*^ *P* < 0.05 versus day 0; ^†^ *P* < 0.05 versus day 3. PCR: polymerase chain reaction;* Agg*: aggrecan;* COL2A1*: collagen type II; SDs: standard deviations.

qRT-PCR revealed marked downregulation of aggrecan gene (*Agg*) expression under static load after culturing compared with fresh tissues (Figure 5, Table 3). In contrast, collagen II (*COL2A1*) expression was upregulated under static load on day 3 and the difference was significant with fresh tissue. However, the* COL2A1 *expression subsequently decreased rapidly to below detectable levels under static load until day 14 (Figure 5 and Table 4).

## 4. Discussion

The initial objective of this study was to investigate the effect of static load on the whole IVD motion segment in culture to establish conditions for the future study of the effect of complex dynamic loading on IVDs. Abnormal mechanical loading plays an important role in the pathogenesis of IVD degeneration [4, 32–34]. Moreover, static loading can lead to alterations in the histological and biochemical properties of the IVDs [16, 35, 36]. However, the IVDs are the key load-bearing components of the spine, and they are responsible for buffering shock, maintaining spinal stability, and distributing external force [37]. Therefore, it is essential to study the biology and mechanobiology of the IVDs to improve the treatment of disc degeneration and LBP.

A suitable model can provide an experimental platform for related research. All models have advantages and shortcomings depending on the specific hypotheses. Because of the lack of close control and monitoring of the mechanical environment of* in vivo* models, it is difficult to examine specific aspects of cell signal transduction and matrix metabolism [1]. The* in vitro* culturing of isolated disc cells is widely performed to analyze specific factors; however, because they are separated from the original ECM, it is difficult to maintain the original cell properties and interactions between cells, leading to the loss of cell phenotypes [38]. Compared with the above methods, culturing of the whole disc organ can enable the maintenance of cell-to-cell interactions, as well as interactions between cells and the ECM, which is conducive to studying the metabolism and degeneration of the discs under controlled conditions. Moreover, this method is convenient for observing the responses of the IVD tissues to specific external stimuli [39–41].

Accordingly, Lim et al. [27] developed an* in vitro* organ culture model of the rat spinal motion segment and demonstrated the maintenance of viability and tissue integrity for 14 days. This spinal motion segment included the VBs-IVDs. In previous studies [11, 15, 42], the loading was applied directly to the surface of the IVD organ, and thus pressure was applied through the cartilage EP or the AF, leading to a specimen loading environment that significantly differed from the actual biological situation.* In vivo* loading is applied to the bone EP through the VBs, and then the cartilage EP is compressed, and the force is transferred to each part of the AF to avoid uneven stress. Finally, it is transferred to the NP [43, 44]. The major difference between our study and previous studies is that we tested the motion segment, which we believe enabled this* ex vivo* IVD organ model to more closely resemble the actual physiological state and thus provided a more accurate simulation of spinal loading conditions. In addition, the adjoining VBs (as rigid fixtures) might facilitate the application and control of complex mechanical loads and thus allow for the further study of IVD biomechanics.

Seol et al. [28] compared rabbit and rat IVD motion segments and found that GAG (glycosaminoglycans) loss was minimal in rabbit IVDs but that it was progressive and severe in rat IVDs. Further, the rat IVDs showed increased expression of MMP-3 and markedly decreased expression of collagen types I and II compared to the rabbit IVDs. Therefore, the rabbit IVDs were more stable in* ex vivo* culture. Accordingly, we used this motion segment as a model in the current mechanobiological study. Seol et al. [28] did not detect any obvious changes in the GAG content in motion segments cultured for two weeks; however, in our study, a significant decrease in proteoglycan gene expression was detected under static load. This result likely occurred because of limited nutrition and the lack of dynamic mechanical loading. Regardless, we administered anticlotting agents before donor death and after tissue harvesting to prevent blood clot formation as previously described [1, 19, 45]. During prolonged culturing, VBs likely compromise the processing of nutrients through the EP channels [46], resulting in decreased biosynthetic activity. In addition, removal of the biomechanical load significantly affected disc cell production by the matrix [21, 47], and maintenance of the matrix content, as well as cell viability, was expected under mechanical load. Therefore, we will take advantage of this model in future studies.

Numerous* ex vivo* studies [9, 41, 48] have been performed using modified culture systems that have aimed to elucidate the effects of IVD biomechanics on the maintenance of tissue structure and function. For example, Ohshima et al. [15] developed a perfusion apparatus to use osmotic stress in lieu of mechanical stress to apply the load experienced by the discs* in vivo*; in addition, Lee et al. [19] designed a chamber based on that of Ohshima et al. for long-term culturing to explore the possibility of the* ex vivo* culturing of large disc explants. However, the results revealed a marked decrease in cell viability after 1 week in the disc EPs. This shortcoming might have been due to the sizes of the discs, given that the organ culturing of large animal IVDs requires systemic anticoagulation [49]. Therefore, Gantenbein et al. [1] used anticoagulation treatments to avoid blood clotting in the EP capillary beds and established a bioreactor to apply diurnal loading to an ovine caudal IVD for 7 days; Jünger et al. [49] improved this culture system by applying low-magnitude cyclic loading in addition to diurnal loading. Further, Chan et al. [23] used a dynamic loading bioreactor to study the effect of more complex loading on disc degeneration. Although the previous devices were applicable for analysis of the IVD organ, they were not suitable for the culturing or loading of the IVD motion segment because the middle disc is softer than both sides of the VBs; moreover, the vertical axis was longer than the IVD organ. The segment was prone to bending deformation under loading, leading to difficult application of vertical compression and easily damaged disc tissues. Therefore, we designed a loading and organ-culturing apparatus for the IVD motion segment. As described previously, the use of jackscrews with pedestals enabled maintenance of the motion segment in an upright position, and the mobile loading plate ensured that full vertical loading was consistently applied on the surface of the model (Figure S2).

In this experiment, the osmolarity of the medium was adjusted to 410 mOsm/kg to control disc swelling [17]. A pilot experiment estimated that the weight can produce a disc compression force of 0.55 ± 0.12 MPa. Studies have shown that the IVDs are subjected to pressure ranging from 0.1 to 1.1 MPa during the day [23, 50] and that compressive loading of over 0.8 MPa could induce early disc degeneration [9, 51]. Because data on physiological loading magnitudes are incomplete for rabbit IVDs, the loading magnitudes applied in this study were based on the physiological range of loading in humans.

Over the two weeks of culturing under constant static compression, the morphological integrity of the IVDs gradually deteriorated after 3 days, and the proteoglycan content of the NP was significantly decreased compared with that in fresh tissue. However, the type II collagen content was obviously increased at 3 days compared with the fresh tissues, after which it was markedly reduced. qRT-PCR analysis also confirmed that* Agg* gene expression was significantly downregulated and significantly different than that prior to culturing; however,* COL2a1* gene expression was upregulated at 3 days, and this trend was consistent with the results of histological and IHC analyses. These phenomena might be explained by Wolff's law [52], which states that an appropriate mechanical load can allow for the maintenance of tissue morphology and can stimulate bone metabolism to synthesize matrix components; however, a persistent or excessive load inhibits these functions. Ohshima et al. [15] assessed the effect of static loading on intact bovine coccygeal discs and found that maximum tissue hydration occurred at a load of 5–10 kg and that lighter (0.5 kg) or heavier loads (15 kg) led to decreased incorporation. In the current experiment, in the presence of intradiscal pressure produced by a load of 3 kg, IHC and RT-PCR analyses revealed that the synthesis of type II collagen was stimulated to resist compression within a brief period (3 days); hence, the type II collagen content was higher compared with those fresh tissues. However, the constant static compression inhibited cell metabolic activity and led to a significant decrease in the collagen content after brief stimulation.

During culture, however, the proteoglycan content did not increase. This finding might have been observed because the static load significantly inhibited the production of proteoglycans. Lee et al. [19] cultured bovine coccygeal discs under a 5 kg static load and found that although NP cell viability was maintained after 1 week, an approximately 50% decrease in proteoglycan synthesis occurred within 2 days of culturing. In contrast, Gantenbein et al. [1] cultured models under cyclic diurnal loading (0.2 MPa for 6 hours and 0.8 MPa for 16 hours) and demonstrated that glycosaminoglycan synthesis was maintained after 7 days, showing that frequent loading can stimulate and maintain proteoglycan synthesis. Under general conditions, the changes in the levels of type II collagen and aggrecan are consistent [10–13, 16–22], but Neidlinger-Wilke et al. [53] found that IVDs under low hydrostatic pressure (0.25 MPa, 30 min, 0.1 Hz) tend to exhibit increased aggrecan expression in cell nuclei and decreased type II collagen expression. Whereas the high hydrostatic pressure tended to decrease the expression of both aggrecan and type II collagen, the author suggested that hydrostatic pressure might regulate disc matrix turnover in a dose-dependent manner. Our experiment further showed that the changes in the two main matrix molecules were inconsistent up to 3 days and that the proteoglycan content was significantly decreased after 3 days but that type II collagen production was briefly stimulated. The differences between our results and those of the above studies are mainly attributed to differences in loading styles; a constant static load can significantly inhibit the production of proteoglycans [17, 19, 24, 54]. In addition, static loading may have stronger effects on proteoglycans than on type II collagen. Thus, a shorter observational period will be used in a future study to test this hypothesis.

Future studies should aim to improve the* ex vivo* culture system and apparatus for application of a consistent physiologic load and complex dynamic load to maintain the functions of the model over a longer duration. In addition, the* ex vivo* model should be used to further examine IVD biomechanics and to observe the means through which degenerative discs can be manipulated to provide a basis for the clinical treatment of degenerative disc diseases.

In conclusion, a loading and organ culture system for* ex vivo* rabbit IVD motion segments was developed. Constant static compression led to IVD degeneration and specifically to a change in the proteoglycan content, which was markedly decreased after culturing. Nevertheless, we found that static compression stimulated the synthesis of type II collagen within a brief time period. Thus, the results of this study indicate that an appropriate mechanical load can stimulate matrix synthesis but that constant static compression leads to progressive disc degeneration.

## Supplementary Material

Figure S1: Detailed view of the specimen culturing conditions.
Figure S2: Loading and organ culturing apparatuses for the rabbit IVD motion segments (diagrammatic sketch).

## Figures and Tables

**Table 1:** Details for the primers for the target and reference genes used in quantitative real-time PCR.

Gene	Primers	Primer sequence	bp
*GAPDH*	tuzi-GAPDH-F	CGAGACACGATGGTGAAGGT	131
	tuzi-GAPDH-R	ATGTAGTGGAGGTCAATGAATGG	
*Tu-COL2A1*	Tu-COL2A1-F	ATGGCGGCTTCCACTTCA	92
	Tu-COL2A1-R	CTCAGTGGACAGCAGGCG	
*Tu-agg*	Tu-agg-F	TTACCACCTACCCTTCACCTG	90
	Tu-agg-R	TTCTTCTGTCCAAAGGTCCTG	

*GAPDH*: glyceraldehyde-3-phosphate dehydrogenase; *COL2A1*: collagen II; *agg*: aggrecan; F: forward; R: reverse.

**Table 2:** Collagen type II IHC staining intensity of the NP during organ culture.

Culture time point	Day 0	Day 3	Day 7	Day 14
Static load	43.75 ± 7.05	61.54 ± 5.91^*∗*^	22.97 ± 5.03^*∗*†^	30.43 ± 2.13^*∗*†^

The values represent the means ± SD. ^*∗*^Significance of differences between culture time point and fresh tissue (day 0); ^†^significance of differences between culture time point and day 3. IHC: immunohistochemistry; NP: nucleus pulposus; SD: standard deviation.

**Table 3:** *Agg *gene expression quantified by real-time PCR during organ culture.

Culture time point	Day 0	Day 3	Day 7	Day 14
Static load	1.000 ± 0.037	0.053 ± 0.060^*∗*^	0.007 ± 0.001^*∗*†^	0.027 ± 0.005^*∗*^

The values represent the means ± SD. ^*∗*^Significance of differences between culture time point and fresh tissue (day 0); ^†^significance of differences between culture time point and day 3. Agg: aggrecan; PCR: polymerase chain reaction; SD: standard deviation.

**Table 4:** *COL2a1 *gene expression quantified by real-time PCR during organ culture.

Culture time point	Day 0	Day 3	Day 7	Day 14
Static load	1.000 ± 0.038	1.195 ± 0.040^*∗*^	0.016 ± 0.002^*∗*†^	0.005 ± 0.001^*∗*†^

The values represent the means ± SD. ^*∗*^Significance of differences between culture time point and fresh tissue (day 0); ^†^significance of differences between culture time point and day 3. COL2a1: collagen type II; PCR: polymerase chain reaction; SD: standard deviation.
